# Colorimetric and Electrochemical Dual-Mode Detection of Thioredoxin 1 Based on the Efficient Peroxidase-Mimicking and Electrocatalytic Property of Prussian Blue Nanoparticles

**DOI:** 10.3390/bios14040185

**Published:** 2024-04-10

**Authors:** Jeong Un Kim, Jee Min Kim, Annadurai Thamilselvan, Ki-Hwan Nam, Moon Il Kim

**Affiliations:** 1 Department of BioNano Technology, Gachon University, 1342 Seongnamdae-ro, Sujeong-gu, Seongnam 13120, Republic of Korea; unhyun123@naver.com (J.U.K.); jeem424@naver.com (J.M.K.); tamilselvan8033@gmail.com (A.T.); 2 Division of Research and Development Equipment Industry, Center for Scientific Instrumentation, Korea Basic Science Institute, 169-148 Gwahak-ro, Yuseong-gu, Daejeon 34133, Republic of Korea

**Keywords:** Prussian blue nanoparticles, dual-mode detection, nanozyme, thioredoxin 1, cancer biomarker

## Abstract

As a potent detection method for cancer biomarkers in physiological fluid, a colorimetric and electrochemical dual-mode sensing platform for breast cancer biomarker thioredoxin 1 (TRX1) was developed based on the excellent peroxidase-mimicking and electrocatalytic property of Prussian blue nanoparticles (PBNPs). PBNPs were hydrothermally synthesized using K_3_[Fe(CN)_6_] as a precursor and polyvinylpyrrolidone (PVP) as a capping agent. The synthesized spherical PBNPs showed a significant peroxidase-like activity, having approximately 20 and 60% lower *K_m_* values for 3,3′,5,5′-tetramethylbenzidine (TMB) and H_2_O_2_, respectively, compared to those of horseradish peroxidase (HRP). The PBNPs also enhanced the electron transfer on the electrode surface. Based on the beneficial features, PBNPs were used to detect target TRX1 via sandwich-type immunoassay procedures. Using the strategies, TRX1 was selectively and sensitively detected, yielding limit of detection (LOD) values as low as 9.0 and 6.5 ng mL^−1^ via colorimetric and electrochemical approaches, respectively, with a linear range of 10–50 ng mL^−1^ in both strategies. The PBNP-based TRX1 immunoassays also exhibited a high degree of precision when applied to real human serum samples, demonstrating significant potentials to replace conventional HRP-based immunoassay systems into rapid, robust, reliable, and convenient dual-mode assay systems which can be widely utilized for the identification of important target molecules including cancer biomarkers.

## 1. Introduction

Breast cancer is the most common cancer in females, with an incidence rate of 31.4% of all female cancer patients in 2022. It is also the cause of 15% of female cancer fatalities [1]. Based on its seriousness, there has been significant interest in early diagnosis as well as monitoring the progress of the disease [2], however, traditional diagnostic methods such as imaging and tissue biopsies are invasive, time-consuming, and may lack the necessary sensitivity for early-stage cancers [3]. Therefore, the development of a low-cost and efficient method for diagnosing breast cancer using physiological fluid like blood is urgently needed [4]. In this regard, liquid biopsy has recently progressed as a minimally invasive diagnostic method for cancer through the quantification of tumor-derived substances, such as circulating tumor cells (CTCs) and circulating tumor-related compounds like nucleic acids and proteins that are collected from body fluids such as peripheral blood [5]. So far, it is considered to be a replacement for existing complicated breast cancer diagnostic methods owing to its ease and efficiency.

TRX1 is a 12 kDa protein that catalyzes the reduction of disulfide bonds in substrate proteins through disulfide reduction activity in vivo, and then contributes on various biological functions including cell proliferation and death. In particular, it has been discovered that TRX1 is closely related with the survival of breast cancer cells [6], and importantly, it is involved in the whole tumor development process, whereas other biomarkers such as carcinoembryonic antigen (CEA) and cancer antigen 15-3 (CA15-3) can only be found in overstated tumors [7,8]. Thus, detection of TRX1 in physiological fluids is now considered a suggestable option for diagnosing breast cancer at an early stage. TRX1 is secreted from cancer cells, resulting in the elevation of its concentration in the blood plasma of cancer patients, which can be strong evidence for the presence of tumors [9,10,11,12]. The most common method used for quantitative analysis of TRX1 is an enzyme-linked immunosorbent assay (ELISA) [13,14,15,16], however, ELISA technique has the limitations of natural enzymes, which include the high costs of production and purification, limited stability, and issues in detection reliability due to the alteration of activity under harsh environmental conditions [17,18]. Therefore, it is highly desirable to develop an innovative detection method enabling more stable, economical, reliable, selective, and sensitive identification of TRX1 in physiological fluids like blood plasma.

Recently, nanomaterials that exhibit enzyme-like properties (nanozymes) have gathered a wide attention as potent alternatives to natural enzymes. When compared to natural enzymes, nanozymes exhibit greater stability and robustness, reduced production costs, easier surface functionalization, and the potential for achieving enhanced catalytic performances through further engineering, along with several unique merits of nanostructures including high conductivity, large surface area, and other physicochemical properties such as magnetism and fluorescence, making nanozymes promising candidates in various analytical and biomedical areas [19,20,21]. Until now, nanozymes can mimic four types of enzyme—oxidoreductase, hydrolase, lyase, and isomerase—and among them, peroxidase in a family of oxidoreductase has been the most widely studied [22]. Representatively, nanozymes composed of gold [23], iron oxide [24], and cerium oxide [25] have been widely demonstrated to have intrinsic peroxidase-like activities, which catalyze the oxidation of chromogenic substrates like TMB in the presence of H_2_O_2_ to produce color signals corresponding to the oxidized substrates and have been widely utilized in diverse assay methods including ELISA. PBNPs, composed of porous coordination polymer PB where Fe^2+^ and Fe^3+^ ions are alternately linked to cyanide ligands, have also been recognized one of the most efficient peroxidase-like nanozymes [26,27,28]. Their catalytic activity was presumed from the abundant Fe ions within their structures having large surface area with high porosity, acting like active sites of natural enzymes [27,29]. Moreover, they have large cavities that allow the introduction of alkali metal ions, such as Na^+^ and K^+^, into the lattice, which can further increase electrical conductivity. This is highly beneficial for the construction of electrochemical assay systems [30,31,32]. These discoveries have led to diverse analytical applications, however, further investigation is required to make them more appropriate in practical utilizations, through the construction of multi-mode sensing platform with self-checking advantages for detecting important analytes with high credibility. 

In this regard, we have developed a colorimetric-electrochemical dual-mode detection platform for TRX1 based on the peroxidase-mimicking and electrocatalytic properties of PBNPs. PBNPs were simply synthesized via a hydrothermal method using the single precursor of K_3_[Fe(CN)_6_] by varying the concentrations of the PVP capping agent to control the size and morphology of PBNPs. The optimized PBNPs were demonstrated to have both high peroxidase-like and electrocatalytic properties. Based on these properties, TRX1 was successfully detected through the construction of sandwich-type immunoassay via both colorimetric and electrochemical modes. Various analytical characteristics such as selectivity, sensitivity, and practical utility with detection precisions were investigated. 

## 2. Experimental Section

### 2.1. Materials

Hydrogen tetrachloroaurate (III) trihydrate (HAuCl_4_∙3H_2_O), sodium citrate, phosphate buffered saline (PBS), potassium ferricyanide (III) (K_3_[Fe(CN)_6_]), PVP, sodium acetate, H_2_O_2_, TMB, 1-ethyl-3-[3-dimethylaminopropyl]carbodiimide (EDC), N-hydroxysulfo-succinimide (sulfo-NHS), ethyl alcohol, human serum, and bovine serum albumin (BSA) were purchased from Sigma-Aldrich (St. Louis, MO, USA). TRX1 antibody, recombinant human TRX1, and human TRX1 ELISA kit were purchased from Abcam (Cambridge, UK). Syringe filter (0.2 μm pore; Millipore, Darmstadt, Germany) was used to purify the reacted solutions. Transparent 96-well plate for conducting colorimetric immunoassay was purchased from Nunc (Roskilde, Denmark). All solutions were prepared with deionized water purified by a Milli-Q Purification System (Millipore, Darmstadt, Germany).

### 2.2. Synthesis and Characterization of PBNPs

PBNPs were synthesized via a hydrothermal method following the previously reported procedures with slight modifications [33,34,35]. First, 110 mg of K_3_[Fe(CN)_6_] and PVP at varied amounts (1.4–3.8 g) were added to 50 mL of HCl (0.1 M) solution, followed by stirring vigorously to obtain a transparent solution. Then, the mixture was heated at 80 °C using a water bath and stirred for one day. Afterwards, it was washed one time with distilled water and two times with ethanol to remove any remaining reactants, and collected by centrifugation at 10,000× *g* for 5 min. The final PBNPs were freeze-dried and stored at 4 °C until use. 

The synthesized PBNPs were analyzed by scanning electron microscopy (SEM) using a Scanning Electron Microscope (Hitachi S-4700, Tokyo, Japan) coupled with energy-dispersive spectrometer (EDS) (Elementar, Vario Macro, Langenselbold, Germany). X-ray diffraction (XRD) and X-ray photoelectron spectroscopy (XPS) were conducted using X-ray diffractometer (D/MAX-2500, Rigaku Corporation, Tokyo, Japan) and XPS reader (Sigma Probe, Thermo Scientific, Madison, WI, USA), respectively. Fourier transform infrared spectroscopy (FT-IR) was performed to elucidate the chemical functional groups of the PBNPs using an FT-IR spectrophotometer (FT/IR-4600, JASCO, Easton, MD, USA). The particle numbers of PBNPs were measured by Nanoparticle Tracking Analyzer (NTA; Particle Metrix, PMX120, Mebane, NC, USA). 

### 2.3. Evaluation of Peroxidase-Mimicking and Electrocatalytic Activity of PBNPs

Peroxidase-like activity of PBNPs was determined by measuring the level of TMB oxidation for 5 min at room temperature (RT) in the reaction buffer (sodium acetate, 0.1 M, pH 4) with TMB (1 mM) and H_2_O_2_ (10 mM). After the reaction, the supernatant was separated via centrifugation (10,000× *g*, 2 min) and used for analyzing the blue color corresponding to the oxidized TMB by measuring the absorbance in a scanning mode or at 652 nm using a microplate reader (Synergy H1, BioTek, Winooski, VT, USA), serviced by the Center for Bionano Materials Research at Gachon University (Seongnam, Republic of Korea). Steady-state kinetic assays were also conducted using TMB and H_2_O_2_ as substrates. All reactions were monitored in a kinetic mode at 652 nm using the microplate reader. The kinetic parameters were calculated using the Lineweaver-Burk plots derived from the Michaelis–Menten equation, known as *v* = *V_max_* × [*S*]/(*K_m_* + [*S*]), where *v* is the initial velocity, *V_max_* is the maximal reaction velocity, [*S*] is the concentration of substrate, and *K_m_* is the Michaelis constant.

Electrocatalytic activity of PBNPs was determined via cyclic voltammetry (CV) using a CHI600E Potentiostat (IJ Cambria Scientific Ltd., Austin, TX, USA). A conventional three-electrode electrochemical system was employed, with PBNPs as the working electrode. The reference electrode was Ag/AgCl saturated in KCl and a platinum wire served as the counter electrode. Typically, PBNPs at different concentrations were deposited on the working electrode using an aqueous PBS (0.1 M) solution containing 0.1 M KCl and 5.0 mM of [Fe(CN)_6_]^3−/4−^ as a redox probe. The sweep rate was set at 100 mV s^−1^, with scanning from −0.4 to 0.6 V. Electron transfer activity according to the scan rate was measured by measuring current signals in a range of 10 to 100 mV s^−1^ on both glassy carbon electrode and screen-printed electrode (SPE; Metrohm DropSens, Oviedo, Spain) using the CV technique. Impedance was also measured at a potential of 10 mV in the frequency range of 100 kHz to 100 MHz, using electrochemical impedance spectroscopy (EIS).

### 2.4. Preparation of PBNPs Conjugated with TRX1 Antibody (PBNPs-Ab)

TRX1 antibody was conjugated on PBNPs via physical adsorption. PBNPs (1 nM calculated by NTA) and TRX1 antibody (10 μg mL^−1^) were mixed in 1 mL of boric acid buffer (0.1 M, pH 6), followed by mixing for 1 h under gentle rotation at RT. Unbound antibodies were removed by washing three times using the same buffer, and the supernatant was used to calculate the antibody conjugation efficiency using a BCA protein assay kit (Thermo Fisher Scientific). 

After the physical conjugation, the PBNPs-Ab were incubated with 1% BSA to block non-specific binding on the surface and stored at 4 °C until use. Surface zeta potentials of PBNPs were measured at different pH conditions using NTA, to set up different charges between PBNPs and TRX1 antibodies.

### 2.5. Dual-Mode Immunoassay for TRX1 Utilizing Peroxidase-like and Electrocatalytic Activities of PBNPs

For the colorimetric TRX1 immunoassay, 100 μL of TRX1 antibody (10 μg mL^−1^) was added to each well of a transparent 96-well plate and stored overnight at 4 °C. After excessive washings with PBS, BSA (1%) solution was added to the wells and incubated overnight at 4 °C to block nonspecific binding sites. After washing out the remaining BSA, 100 μL of sample solution containing TRX1 at different concentrations (0 to 2 μg mL^−1^) was added to the wells and incubated for 2 h at 37 °C. After washings, 100 μL of PBNPs-Ab was added and allowed antigen-antibody interaction for 1 h at 37 °C. After removing the unbound PBNPs-Ab by washings, the peroxidase-mediated colorimetric reaction was performed for 10 min at RT. Finally, the blue color intensities from the oxidized TMB were measured using the microplate reader. 

Electrochemical TRX1 immunoassay was performed on SPE. To enhance the antibody immobilization efficiency, gold nanoparticles (AuNPs) were first functionalized on the surface of SPE. To this, AuNPs having carboxyl moieties with an average diameter of approximately 40 nm were synthesized by sodium citrate-based reduction method according to a reported method [36]. For the electrochemical assay, 5 μL of AuNPs were dropped on the working electrode and vacuum-dried at RT to modify the surface for efficiently immobilizing capture TRX1 antibody. An amount of 10 μL of MES buffer solution containing EDC (50 mM) and sulfo-NHS (10 mM) was added and incubated for 30 min at RT on the electrode surface. After washing with PBS, 10 μL of TRX1 antibody (10 μg mL^−1^) solution was added and incubated for 1 h at RT. After washing the unbound antibody, 10 μL of BSA (0.3%) was added and incubated for 30 min at RT to block non-specific binding sites. After washing with PBS, 10 μL of sample solution containing TRX1 at different concentrations (0 to 1 μg mL^−1^) was added on the electrode and incubated for 1 h at 37 °C. After washings, 10 μL of PBNPs-Ab was added and allowed antigen-antibody interaction for 1 h at 37 °C. After removing the unbound PBNPs-Ab by washings, CV measurements were conducted as described above.

For quantitatively determining TRX1 in human serum, we confirmed that the initial TRX1 concentration in human serum was negligible using commercial ELISA kit. Then, predetermined amounts of TRX1 were added to human serum to make spiked serum. Finally, the concentration of TRX1 in each spiked sample was determined using the same procedures described above. Recovery rate [recovery (%) = measured value/added value × 100] and the coefficient of variation [CV (%) = SD/average × 100] were determined to evaluate the precision and reproducibility of both colorimetric and electrochemical assays. 

## 3. Results and Discussion

### 3.1. Synthesis and Characterization of PBNPs

**Scheme 1:**
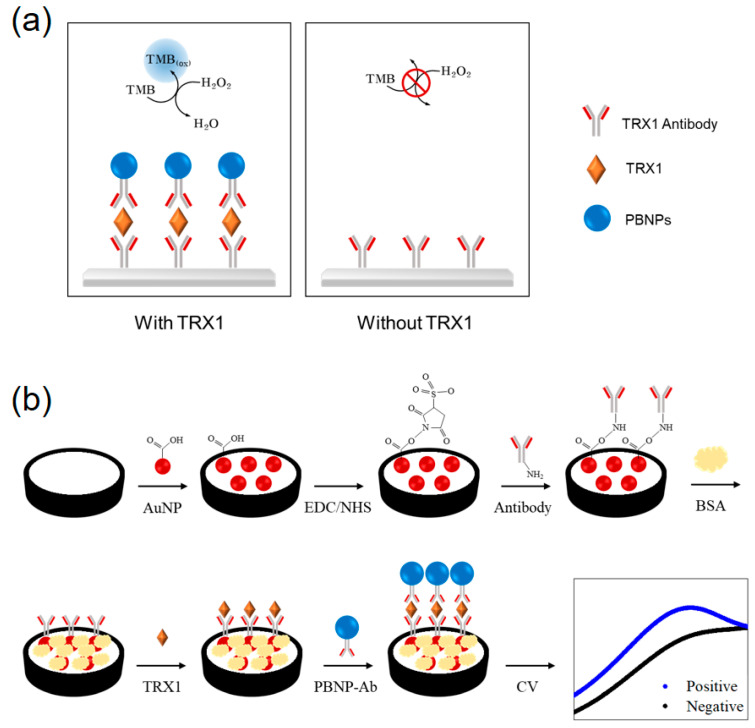
Schematic illustration of PBNPs-based dual-mode immunoassay enabling (**a**) colorimetric and (**b**) electrochemical detection of TRX1.

We hypothesized that PBNPs, which can be easily synthesized by hydrothermally reducing a precursor K_3_[Fe(CN)_6_] and adding the right amount of PVP capping agent to optimize their size and morphology, could be an effective signaling agent that allows for both colorimetric and electrochemical dual-mode detection in the immunoassay for the cancer biomarker TRX1. It has been demonstrated that PBNPs exhibit peroxidase-like and electrocatalytic properties [37,38,39,40,41]. By using sandwich-type immunoassay in the presence of TRX1, PBNPs-Ab are anticipated to bind target antigen in samples. This process produces colorimetric and electrocatalytic signals that are proportional to the quantity of target TRX1 via the action of peroxidase-like activity that induces the oxidation of TMB to produce blue color and electrocatalytic activity to facilitate electron transfer, consequently yielding selective and sensitive identification of TRX1 with high credibility (Scheme 1). 

PBNPs were synthesized using a hydrothermal reduction of single precursor K_3_[Fe(CN)_6_] with PVP capping agent (Figure S1). In the absence of PVP, non-uniform and relatively bigger PBNPs were formed, proving the essential role of PVP in the synthesis of PBNPs (Figure S2). As the employed concentration of PVP increased, the size of PBNPs decreased by retarding the reduction process, and moreover, their shape changed from cube to sphere (Figure S3a). Interestingly, among diverse PBNPs synthesized using different amounts of PVP (1.4, 2.2, 3.0, and 3.8 g in 50 mL reaction solution), the PBNPs prepared at 1.4 g PVP yielded the best peroxidase-like activity (Figure S3b,c), presumably due to its enlarged surface area by smaller size and spherical morphology. Based on this, spherical PBNPs were chosen for further studies. 

**Figure 1:**
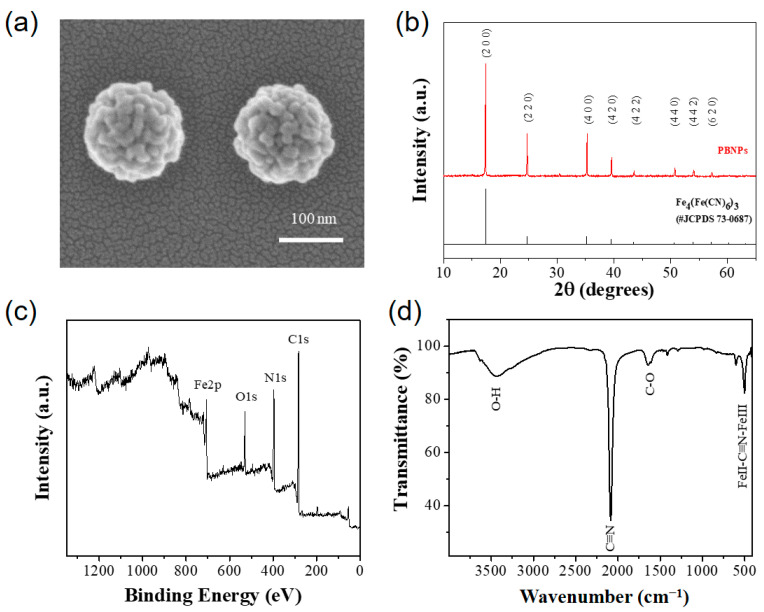
Characterization of PBNPs. (**a**) SEM image, (**b**) XRD, (**c**) XPS survey, and (**d**) FT-IR analysis.

PBNPs were analyzed via SEM, XRD, XPS, EDS, and FT-IR analyses. The optimized spherical PBNPs had uniform size of approximately 130 nm (Figure 1a and Figure S3a). The XRD patterns of PBNPs show strong peaks at 17.4° (200), 24.7° (220), 35.2° (400), and 39.5° (420), which are in good consistency with the standard PBNPs (JCPDS No. 73-0687) with Fe_4_[Fe(CN)_6_]_3_ structure (Figure 1b) [42]. XPS spectra show the presence of C, N, O, and Fe in PBNPs (Figure 1c). The C 1s spectrum was deconvoluted into two small peaks at 283.5 and 285.2 eV, which correspond to C-C and C≡N, respectively (Figure S4a). N 1s spectrum also shows two characteristic peaks at 395.9 and 398.2 eV, both of which show the presence of C≡N corresponding to the cyanide ligand (Figure S4b). Fe 2p spectrum shows the peaks situated at 706.9 eV and 720.5 eV belonging to bivalent iron (Fe^2+^), and the peaks at 710.3 and 723.1 eV are attributed to trivalent iron (Fe^3+^) (Figure S4c) [43]. EDS mapping proves the presence of Fe, C, and N species, which were well distributed throughout the PBNPs (Figure S5). In FT-IR analysis, high-intensity absorption peak appeared at 2090 and 500 cm^−1^, showing the stretching mode of C≡N linkers and FeII-C≡N-FeIII network, respectively [44,45]. These characterizations confirmed the successful synthesis of PBNPs having standard composition, chemical bonding, and crystal structure. 

### 3.2. Investigation of Peroxidase-like and Electrocatalytic Activity of PBNPs

**Figure 2:**
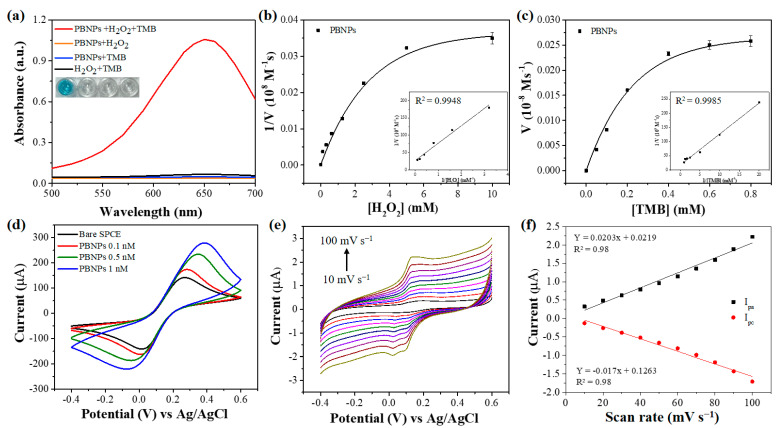
Investigation for peroxidase-like (**a**–**c**) and electrocatalytic (**d**–**f**) activity of PBNPs. (**a**) Absorption spectra of TMB oxidation. Michaelis–Menten curves with Lineweaver–Burk plots of PBNPs toward (**b**) H_2_O_2_ and (**c**) TMB as a substrate. (**d**) Cyclic voltammograms as a function of immobilized concentration of PBNPs on glassy carbon electrode. (**e**) Scan rates study and (**f**) corresponding calibration plots using SPE.

Since the PBNPs have abundant Fe^2+^ and Fe^3+^ ions with large surface area as well as a high cavity to entrap other alkali metal ions to enhance electron transfer, they are expected to have high peroxidase-like and electrocatalytic activity [29,30,31]. To demonstrate this, the peroxidase-like activity of PBNPs was examined by measuring the oxidation of TMB in the presence of H_2_O_2_. The results show that the PBNPs have clear peroxidase-like activity, whereas the other controls that lack one of the reaction components (TMB or H_2_O_2_) did not yield any considerable signal (Figure 2a). The catalytic activity of PBNPs was highly dependent on assay pH conditions, and pH 4 was chosen as the optimal condition for further studies (Figure S6). To elucidate the peroxidation-like action, steady-state kinetic assays were performed using TMB and H_2_O_2_ as substrates (Figure 2b,c). From the Michaelis–Menten curve and the corresponding Lineweaver–Burk plot, *K_m_* values were determined to be 0.36 and 1.4 mM, for TMB and H_2_O_2_, respectively, which are approximately 20 and 60% lower than those of HRP (Table S1). These investigations indicate that the optimized PBNPs can be a potential peroxidase mimic preserving enhanced affinity toward the substrates. 

PBNPs have been widely applied in diverse electrochemical applications due to their low redox potential and high electrocatalytic properties [38]. To examine this, electrocatalytic activity of the PBNPs was investigated. In the CV of the bare SPE without PBNPs in PBS solution containing [Fe(CN)_6_]^3−/4−^, general reversible redox peaks were observed at 0.02 and 0.27 V, and the reduction peak current value was approximately 141 μA (Figure 2d). When PBNPs at 0.1, 0.5, and 1 nM were immobilized on the SPE, the redox peaks shifted and their current values increased to approximately 173, 234, and 279 μA, respectively, indicating that PBNPs accelerated the electron transfer on the electrode. In addition, EIS measurements clearly showed that the resistance of the electrode decreased as the employed concentrations of PBNPs increased, confirming the facilitated electron transfer by the incorporation of PBNPs (Figure S7). Electrochemical performance of PBNPs was investigated by varying the scan rate from 10 to 100 mV s^−1^, with 0.1 M KCl electrolyte in 0.1 M PBS (Figure 2e,f). The results show that the redox currents in the CV rose proportionally depending on the scan rate, and the calibration curves of the oxidation and reduction peaks were linear, indicating that the electrocatalytic reaction by PBNPs follows a surface-controlled process.

PBNPs are expected to be more stable and robust than their enzyme counterpart since they are free from protein backbones. For exact comparison of stability between PBNPs and HRP, experiments were performed to evaluate their stabilities in ranges of pH (1–11), temperature (4–80 °C), and storage time for two weeks at RT (Figure S8). The results show that PBNPs vividly preserved most of their initial activity over 90% in all employed conditions, whereas HRP showed a rapid decrease in acidic pH below 4, high temperature above 50 °C, and storage for over one week. This study demonstrates enhanced stability of PBNPs, which is beneficial to realize their practical applications.

### 3.3. PBNPs-Based Colorimetric and Electrochemical Dual-Mode Immunoassay for TRX1

Based on the peroxidase-like and electrocatalytic properties of PBNPs, immunoassay for TRX1 was developed enabling colorimetric and electrochemical dual-mode detection. First, PBNPs was conjugated with TRX1 antibody via physical adsorption. The immobilization efficiencies of antibody were optimized among several buffers and pHs, and boric acid buffer at pH 6 yielded the best immobilization efficiency of approximately 90% (Figure S9a). At the condition, the surface charge of PBNPs was highly negative (−22 mV) while TRX1 antibody has positive charge considering its isoelectric point (Figure S9b). Thus, electrostatic attraction could be present between PBNPs and TRX1 antibody at pH 6, which might facilitate the preparation of TRX1-Ab. 

**Figure 3:**
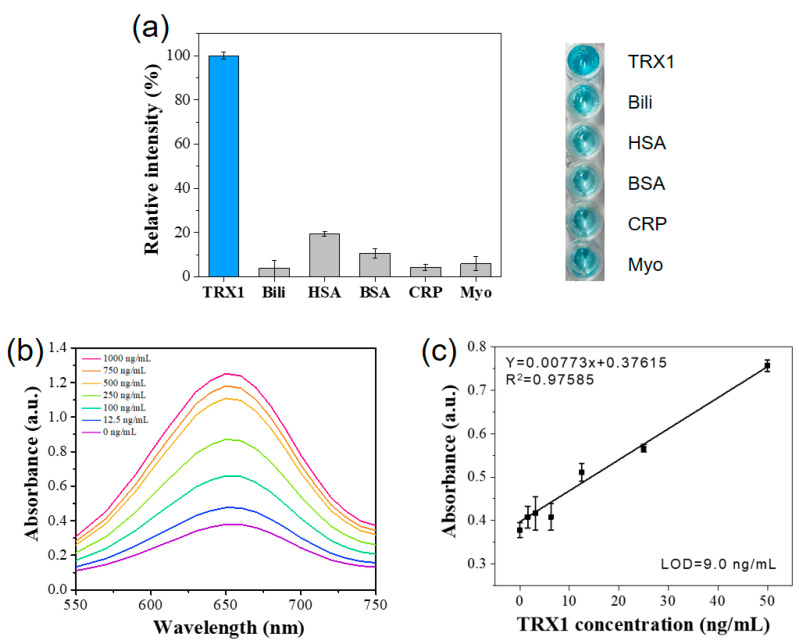
Selectivity and sensitivity for PBNPs-based colorimetric immunoassay for TRX1. (**a**) Absorption intensity and corresponding real photographs for showing selectivity toward TRX1. (**b**) Absorption spectra for the colorimetric detection of TRX1 at diverse concentrations and (**c**) their corresponding linear calibration plots.

Using the TRX1-Ab conjugate, colorimetric immunoassay for TRX1 was constructed. Through the sandwich-type procedures, target TRX1 was selectively detected via the generation of the blue color corresponding to the oxidized TMB, whereas other controls such as bilirubin (Bili), human serum albumin (HSA), bovine serum albumin (BSA), C-reactive protein (CRP), and myoglobin (Myo) did not yield any meaningful response (Figure 3a). The immunoassay system also exhibited linear detection capability, showing increased absorbance intensity as the concentration of TRX1 increased (Figure 3b). Based on the linear calibration plots, the limit of detection (LOD) value was determined to be 9.0 ng mL^−1^ with a linear range from 10 to 50 ng mL^−1^ (Figure 3c). These values are sufficient to detect TRX1 in human body fluid and lower than those of recently reported values in diverse TRX1 detections [13]. 

**Figure 4:**
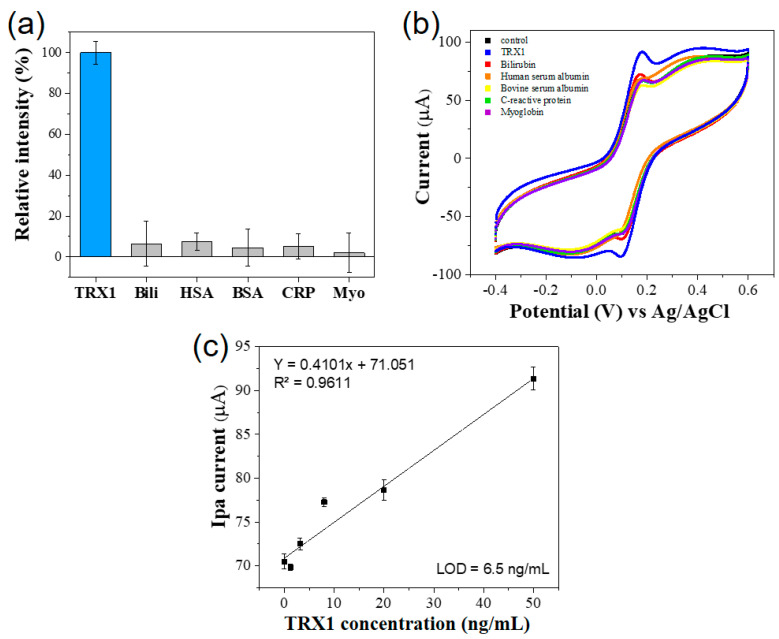
Selectivity and sensitivity for PBNPs-based electrochemical immunoassay for TRX1. (**a**) Selectivity toward TRX1 and (**b**) its corresponding cyclic voltammograms. (**c**) Linear calibration plots to show TRX1 sensitivity.

Electrochemical immunoassay for TRX1 was performed next. AuNPs were first functionalized on the electrode surface to enhance the immobilization efficiency of capture antibody, and other procedures followed the typical sandwich-type immunoassay. As a result, TRX1 was selectively detected via the generation of specific oxidation peak intensity at 0.18 V using CV method, whereas other interfering molecules did not induce any meaningful current signal (Figure 4a,b). Based on the linear calibration plots, the LOD value was determined to be approximately 6.5 ng mL^−1^, which is lower than that of colorimetric immunoassay, with a linear range of 10–50 ng mL^−1^ (Figure 4c). The quantitative performance of PBNPs-based electrochemical immunoassay also fulfilled the current diagnostic cut-off values for TRX1 [13], and thus, has potential to be used practically in diagnostic areas for breast cancer. 

Finally, to investigate the practical applicability of the developed system, the PBNPs-based colorimetric/electrochemical dual-mode immunoassay was applied for quantitative determination of TRX1 spiked in human serum. The spiked levels of TRX1 in human serum for breast cancer diagnosis were selected as: normal group, 10 ng mL^−1^; intermediate group, 25 ng mL^−1^; and breast cancer patient group, 50 ng mL^−1^ [13]. As a result, the assay can quantify TRX1 in human serum with good accuracy and reproducibility. The colorimetric detection yielded CVs and recovery rates of 2.5–7.0% and 99.6–106.0%, respectively, while the electrochemical one yielded CVs and recovery rates of 2.9–6.5% and 92.4–99.3%, respectively (Table 1). These results demonstrate that the PBNPs-based dual-mode immunoassay can be used as a reliable analytical system for detecting TRX1 in clinical samples.

## 4. Conclusions

We demonstrated that PBNPs, optimized by controlling the employed amount of PVP, show both peroxidase-like and electrocatalytic properties, presumably due to the presence of abundant Fe ions, large surface area, as well as sufficient cavity to entrap alkali metals for facilitating electron transfer. PBPNs also showed excellent stability in ranges of pH, temperature, and storage time. Based on the affirmative properties, PBNPs were utilized as a signaling probe inducing both colorimetric and electrochemical reactions in the TRX1 immunoassay. The developed PBNPs-based dual-mode immunoassay enabled successful determination of target TRX1 with high detection precision, selectivity, and sensitivity, which fulfills the clinical cut-off values. Since both detection methods showed excellent analytical performances for TRX1, the dual-mode detection could improve the credibility of diagnosis by efficiently resolving the false positives and false negatives. The proposed assay provides a great potential to develop nanozyme-based diagnostics in practical applications, based on the unique properties of nanozymes. 

## Figures and Tables

**Table 1:** Detection precision of PBNPs-based colorimetric/electrochemical dual-mode immunoassay for TRX1 spiked in human serum samples.

Detection Mode	Spiked Level (ng mL^−1^)	Measured (ng mL^−1^) ^a^	SD ^b^	Recovery (%) ^c^	CV (%) ^d^
Colorimetric method	10	10.57	0.73	105.70	6.96
	25	26.48	1.65	105.92	6.25
	50	49.82	1.01	99.64	2.53
Electrochemical method	10	9.93	0.29	99.26	2.92
	25	24.53	0.69	98.13	3.53
	50	46.22	2.96	92.44	6.42

^a^ Mean of three independent measurements. ^b^ Standard deviation. ^c^ Measured value/expected value × 100. ^d^ Coefficient of variation.

## Data Availability

Data are contained within the article.

## Supplementary Materials

The following supporting information can be downloaded at: https://www.mdpi.com/article/10.3390/bios14040185/s1, Figure S1: Schematic illustration for the synthesis of PBNPs having different sizes and morphologies; Figure S2: SEM images of PBNPs synthesized (a) in the absence and (b) presence of PVP; Figure S3: (a) SEM images and the statistical size analysis of PBNPs and (b) their absorption spectra for TMB oxidation in the presence of H_2_O_2_ with (c) corresponding photographs; Figure S4: High-resolution XPS spectra for (a) C 1s, (b) N 1s, and (c) Fe 2p; Figure S5: (a) SEM image and EDS maps of (b) Fe, (c) C, and (d) N of PBNPs; Figure S6: Effects of pH on the peroxidase-like activity of PBNPs; Figure S7: Nyquist impedance plots as a function of employed concentrations of PBNPs on the electrode; Figure S8: Comparison of stability in ranges of (a) pH, (b) temperature, and (c) storage time at RT; Figure S9: (a) Comparison of TRX1 antibody encapsulation efficiency in different buffers and pH conditions. (b) Zeta potential of PBNPs as a function of buffer pH with predicted isoelectric point of TRX1 antibody; Table S1: Comparison of kinetic parameters of PBNPs with those of recent peroxidase-like nanozymes. References [46,47,48,49,50,51,52,53] are cited in the Supplementary Materials.
