# Supplementary material for: Colorimetric and Electrochemical Dual-Mode Detection of Thioredoxin 1 Based on the Efficient Peroxidase-Mimicking and Electrocatalytic Property of Prussian Blue Nanoparticles
Source: Biosensors (Basel). 2024 Apr 10;14(4):185. doi: 10.3390/bios14040185 (PMC11047952; doi:10.3390/bios14040185)
Supplement: Supplementary file 1 [file biosensors-14-00185-s001.zip › biosensors-2935079-supplementary.pdf]

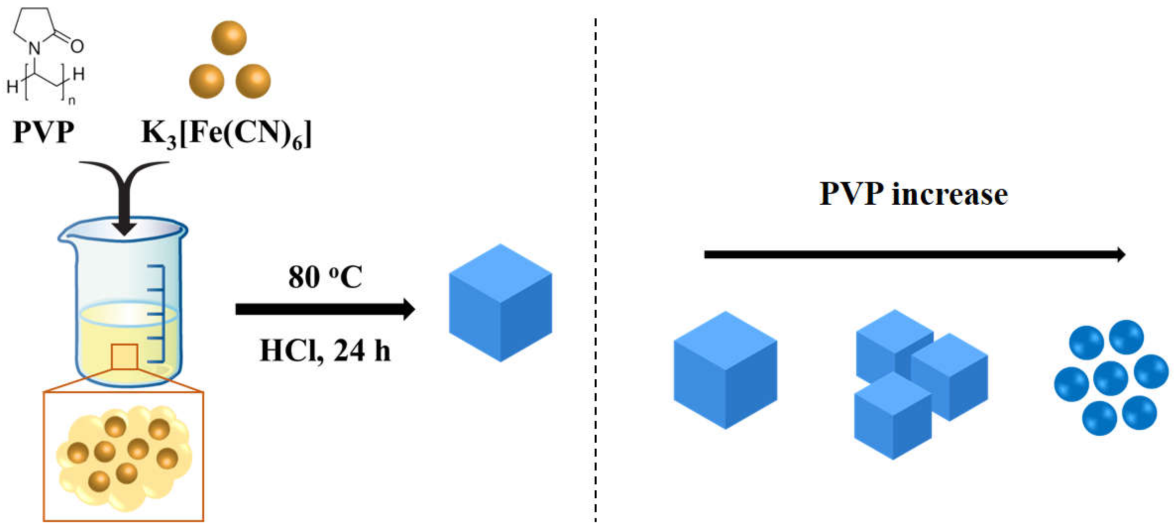

Figure S1. Schematic illustration for the synthesis of PBNPs having different sizes and morphologies.

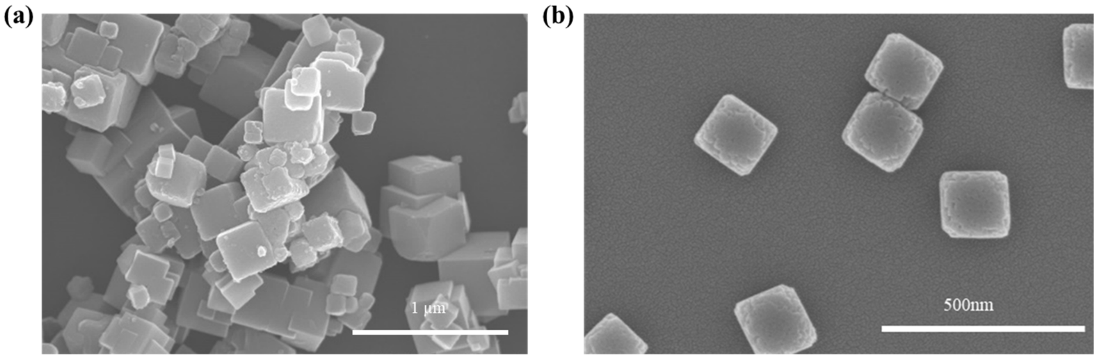

Figure S2. SEM images of PBNPs synthesized (a) in the absence and (b) presence of PVP.

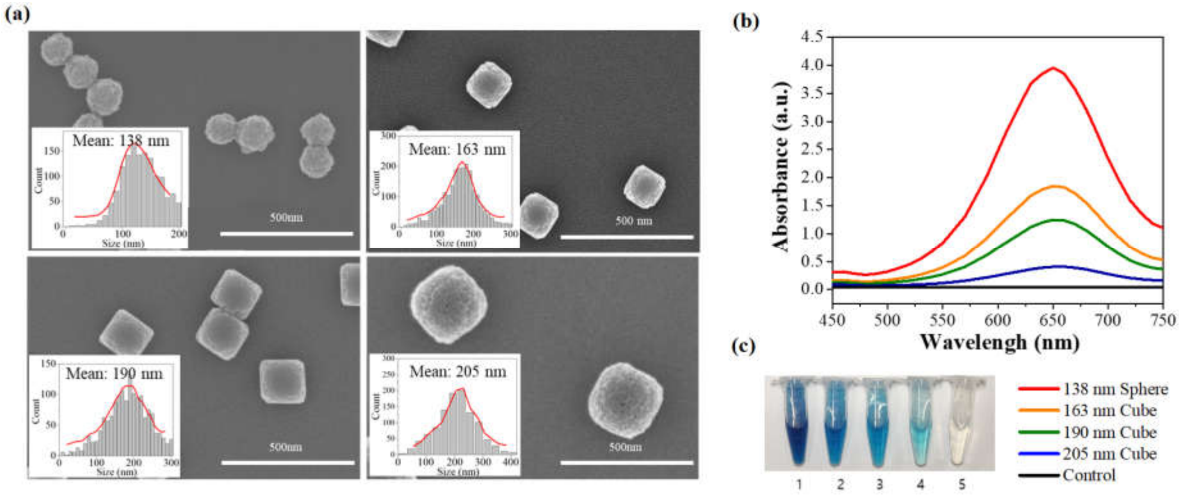

Figure S3. (a) SEM images and the statistical size analysis of PBNPs and (b) their absorption spectra for TMB oxidation in the presence of H_2O_2 with (c) corresponding photographs.

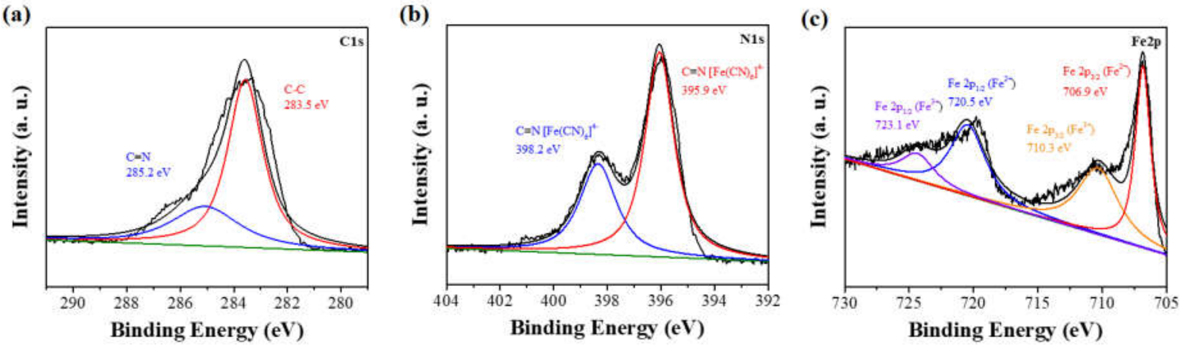

Figure S4. High-resolution XPS spectra for (a) C 1s, (b) N 1s, and (c) Fe 2p.

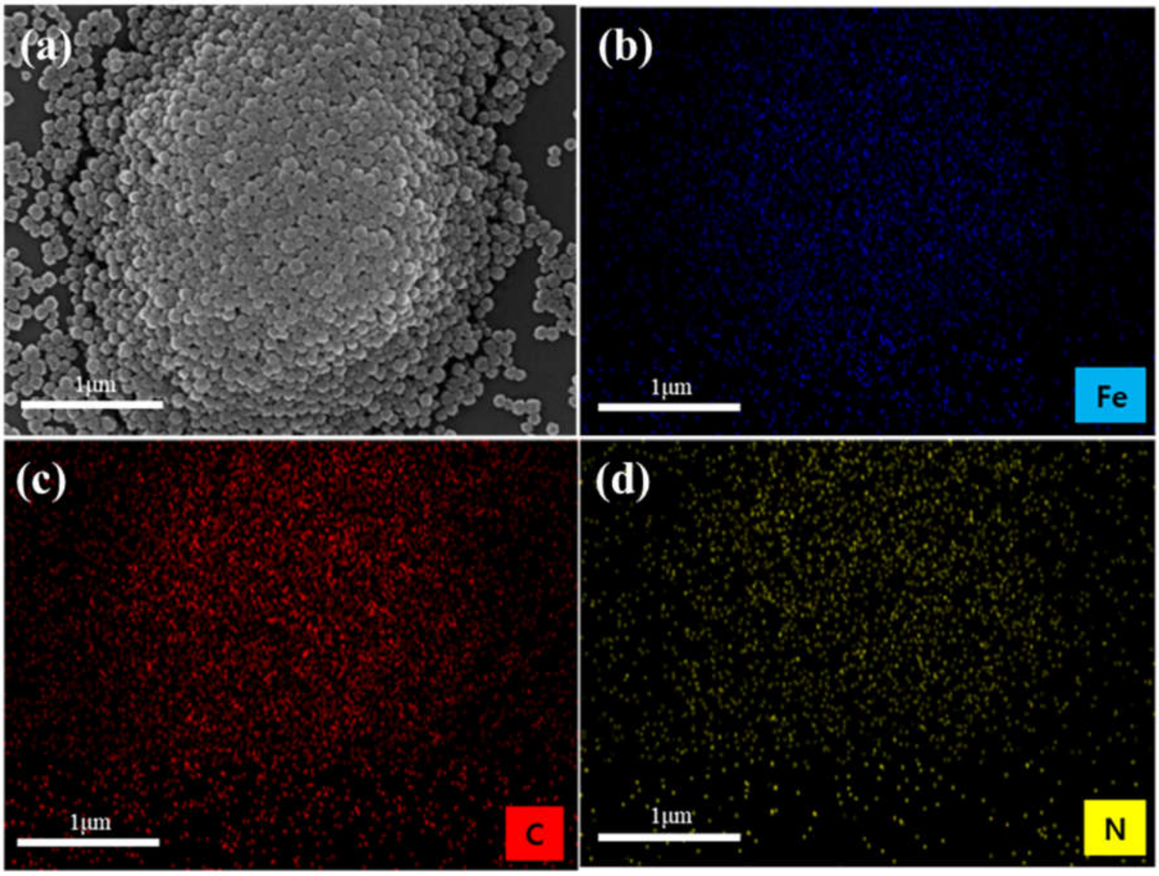

Figure S5. (a) SEM image and EDS maps of (b) Fe, (c) C, and (d) N of PBNPs.

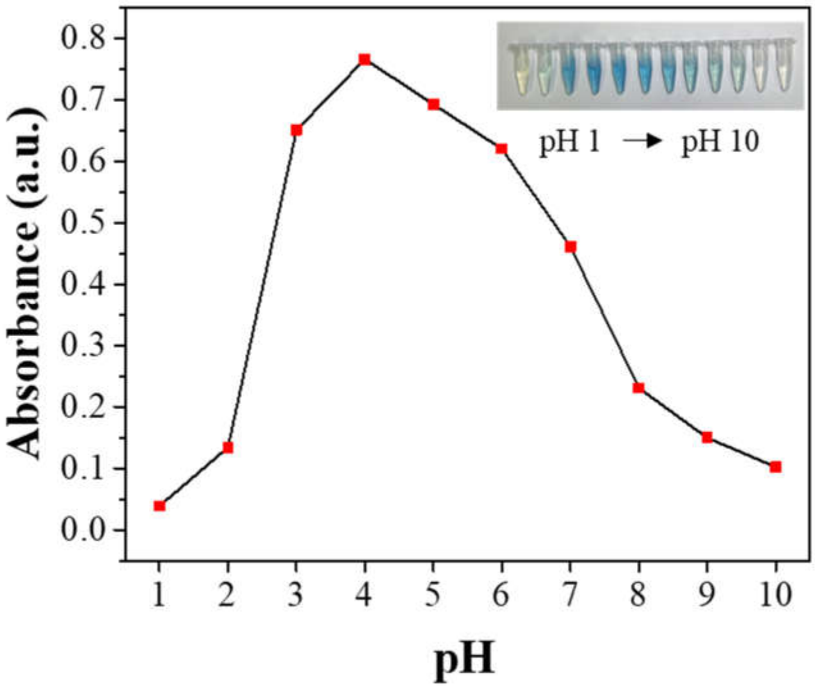

Figure S6. Effects of pH on the peroxidase-like activity of PBNPs.

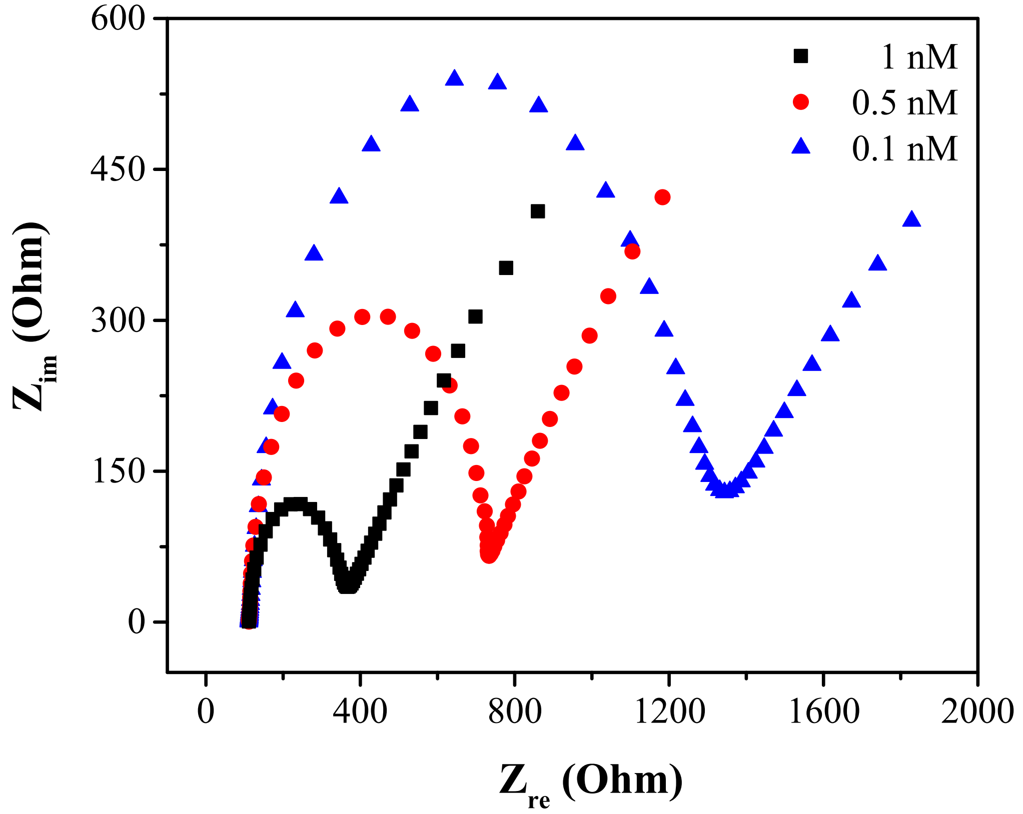

Figure S7. Nyquist impedance plots as a function of employed concentrations of PBNPs on the electrode.

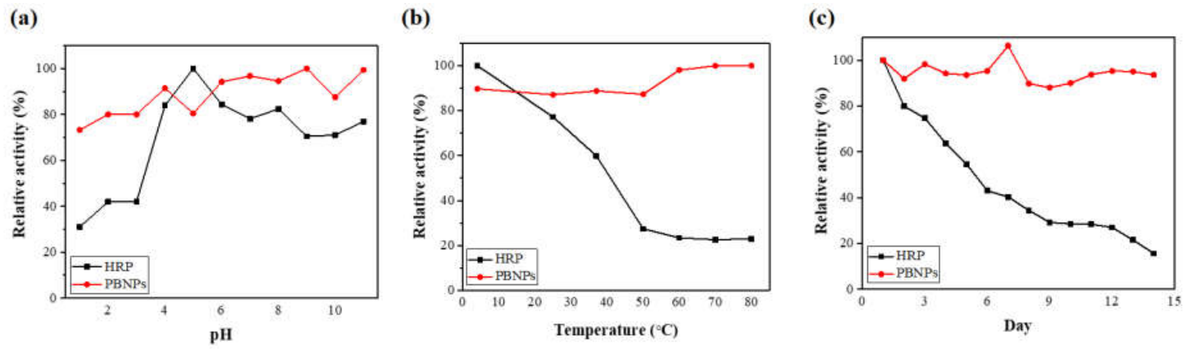

Figure S8. Comparison of stability in ranges of (a) pH, (b) temperature, and (c) storage time at RT.

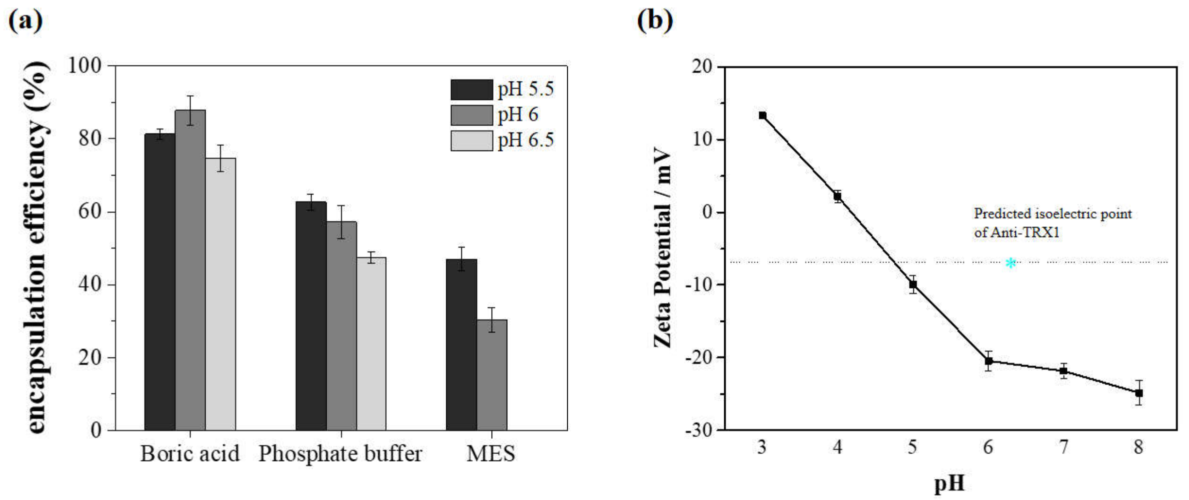

Figure S9. (a) Comparison of TRX1 antibody encapsulation efficiency in different buffers and pH conditions. (b) Zeta potential of PBNPs as a function of buffer pH with predicted isoelectric point of TRX1 antibody.

Table S1. Comparison of kinetic parameters of PBNPs with those of recent peroxidase-like nanozymes.

Catalyst	K_m (mM)		V_{max} ($M\ s^{-1}$)		Ref.
	TMB	H_2O_2	TMB	H_2O_2	
**HRP**	0.434	3.7	1.00×10^{-7}	8.70×10^{-8}	[2]
**Fe_3O_4**	0.098	154	3.44×10^{-8}	9.78×10^{-8}	[2]
**Co_3O_4**	0.037	140	6.27×10^{-8}	1.21×10^{-7}	[3]
**6Fe/CeO_2**	0.176	47.6	8.60×10^{-8}	1.66×10^{-7}	[4]
**Fe-MIL-88B-NH_2**	2.6	1.3	5.60×10^{-8}	2.50×10^{-8}	[5]
**Fe-MOFs**	0.31	0.17	4.00×10^{-8}	1.70×10^{-8}	[6]
**MoS_2-CPBNPs**	0.22	3.17	6.36×10^{-8}	1.49×10^{-8}	[7]
**Cu@PB NCs**	0.6	0.14	1.79×10^{-7}	1.20×10^{-7}	[8]
**PB NCs**	0.8	1.58	9.90×10^{-8}	9.70×10^{-8}	[8]
**PBNPs**	0.363	1.4	1.30×10^{-7}	2.46×10^{-7}	This work

References

1. Lee, M.J.; Lee, E.S.; Kim, T.H.; Jeon, J.W.; Kim, Y.; Oh, B.K. Detection of thioredoxin-1 using ultra-sensitive ELISA with enzyme-encapsulated human serum albumin nanoparticle. Nano Converg. 2019, 6, 1-7.
2. Gao, L.; Zhuang, J.; Nie, L.; Zhang, J.; Zhang, Y.; Gu, N.; Wang, T.; Feng, J.; Yang, D.; Perrett, S.; et al. Intrinsic peroxidase-like activity of ferromagnetic nanoparticles. Nat. Nanotechnol. 2007, 2, 577-583.
3. Mu, J.; Wang, Y.; Zhao, M.; Zhang, L. Intrinsic peroxidase-like activity and catalase-like activity of Co 3 O 4 nanoparticles. Chem. Commun. 2012, 48, 2540-2542.
4. Jampaiah, D.; Reddy, T.S.; Kandjani, A.E.; Selvakannan, P.; Sabri, Y.M.; Coyle, V.E.; Shukla, R.; Bhargava, S.K. Fe-doped CeO 2 nanorods for enhanced peroxidase-like activity and their application towards glucose detection. J. Mat. Chem. B 2016, 4, 3874-3885.
5. Xu, W.; Jiao, L.; Yan, H.; Wu, Y.; Chen, L.; Gu, W.; Du, D.; Lin, Y.; Zhu, C. Glucose oxidase-integrated metal–organic framework hybrids as biomimetic cascade nanozymes for ultrasensitive glucose biosensing. ACS Appl. Mater. Interfaces 2019, 11, 22096-22101.
6. Chen, J.; Gao, H.; Li, Z.; Li, Y.; Yuan, Q. Ferriporphyrin-inspired MOFs as an artificial metalloenzyme for highly sensitive detection of H2O2 and glucose. Chin. Chem. Lett. 2020, 31, 1398-1401.
7. Zhu, Z.; Gong, L.; Miao, X.; Chen, C.; Su, S. Prussian Blue Nanoparticle Supported MoS2 Nanocomposites as a Peroxidase-Like Nanozyme for Colorimetric Sensing of Dopamine. Biosensors 2022, 12, 260.
8. Fan, S.; Jiang, X.; Yang, M.; Wang, X. Sensitive colorimetric assay for the determination of alkaline phosphatase activity utilizing nanozyme based on copper nanoparticle-modified Prussian blue. Anal. Bioanal. Chem. 2021, 413, 3955-3963.
